# Predicting zinc binding at the proteome level

**DOI:** 10.1186/1471-2105-8-39

**Published:** 2007-02-05

**Authors:** Andrea Passerini, Claudia Andreini, Sauro Menchetti, Antonio Rosato, Paolo Frasconi

**Affiliations:** 1 Machine Learning and Neural Networks Group, Dipartimento di Sistemi e Informatica, Università degli Studi di Firenze, Italy; 2 Magnetic Resonance Center (CERM) and Dipartimento di Chimica, Università degli Studi di Firenze, Italy

## Abstract

**Background:**

Metalloproteins are proteins capable of binding one or more metal ions, which may be required for their biological function, for regulation of their activities or for structural purposes. Metal-binding properties remain difficult to predict as well as to investigate experimentally at the whole-proteome level. Consequently, the current knowledge about metalloproteins is only partial.

**Results:**

The present work reports on the development of a machine learning method for the prediction of the zinc-binding state of pairs of nearby amino-acids, using predictors based on support vector machines. The predictor was trained using chains containing zinc-binding sites and non-metalloproteins in order to provide positive and negative examples. Results based on strong non-redundancy tests prove that (1) zinc-binding residues can be predicted and (2) modelling the correlation between the binding state of nearby residues significantly improves performance. The trained predictor was then applied to the human proteome. The present results were in good agreement with the outcomes of previous, highly manually curated, efforts for the identification of human zinc-binding proteins. Some unprecedented zinc-binding sites could be identified, and were further validated through structural modelling. The software implementing the predictor is freely available at:

**Conclusion:**

The proposed approach constitutes a highly automated tool for the identification of metalloproteins, which provides results of comparable quality with respect to highly manually refined predictions. The ability to model correlations between pairwise residues allows it to obtain a significant improvement over standard 1D based approaches. In addition, the method permits the identification of unprecedented metal sites, providing important hints for the work of experimentalists.

## Background

Knowledge about the capability to bind metal ions is important when investigating the function of an experimentally uncharacterized protein. Unfortunately, the identification of bound metal ions can be quite difficult experimentally, especially when attempted at the whole proteome scale. Some results in this direction (metalloproteomics) have been recently reported [1-3], but these techniques are still far from becoming available for routine application. Furthermore, experimental approaches may suffer from biases such as incorporation of the wrong metal cofactor *in vivo*, removal of the metal ion(s) during protein purification procedures, binding of metals at adventitious sites [4]. Within this frame, bioinformatics tools are thus important to guide in the design and in the interpretation of experiments. The prediction of metal binding capabilities is a challenging task for which the development of reliable tools is still in progress [5].

In this paper, we investigate the use of machine learning approaches to automatically annotate metal-binding proteins on the whole-proteome scale. In particular, we focus on an important class of structural and functional sites involving the binding of zinc ions. Zinc is essential for Life and is the second most abundant transition metal ion in living organisms after iron. In contrast to other transition metal ions, such as copper and iron, zinc(II) does not undergo redox reactions thanks to its filled d-shell. In Nature, it has essentially two possible roles: catalytic or structural [6,7]. In humans, zinc has a crucial importance in the complex network of inter-molecular interactions responsible for the proper regulation of protein expression. Indeed, a major role of zinc is in the stabilization of the structure of a huge number of transcription factors such as zinc fingers, which constitute a significant share of the human proteome [8,9]. Only a subset of the natural amino acids can coordinate zinc ions with their side chains. In addition, the binding sites are locally constrained by the requirements on the side chain geometry imposed by coordination chemistry. For these reasons, several sites can be identified with high precision by mining regular expression patterns along the protein sequence while simultaneously inspecting amino acid conservation near the (putative) site [10]. A potential problem with the use of regular expression patterns is that they are usually quite specific but may give a low coverage (many false negatives). On the other hand, a support vector machine (SVM) predictor based on multiple alignments outperforms a predictor based on PROSITE [11] patterns in discriminating between cysteines bound to prosthetic groups and cysteines involved in disulfide bridges [12].

The application of a similar approach to the prediction of zinc-binding properties is not straightforward because most supervised learning algorithms (including SVM) build upon the assumption that examples are sampled independently. Unfortunately, this assumption can be violated when formulating prediction of metal binding sites as a traditional 1D prediction problem. The autocorrelation between the metal binding state is a consequence of the fact that most binding sites contain at least two coordinating residues with short sequence separation. Autocorrelation problems have been recently identified in the context of relational learning [13] and collective classification solutions have been proposed based on probabilistic learners [14,15]. In a recent work [16] we tried to address the autocorrelation problem in the context of metal binding site prediction by developing a two stage approach, where a bi-recurrent neural network refines residue-level SVM predictions by jointly considering all SVM outputs from residues in the same chain when computing the refined prediction for each residue. While the approach performs better then the local SVM predictor alone, such improvement is still not statistically significant. In this work we followed a different approach which aims at exploiting the regularities of zinc-binding sites in terms of sequence separation between ligands. The use of information on the sequential distance between cysteines was recently shown to improve performance in the task of disulfide connectivity prediction [17]. Our solution is based on a reformulation of the learning problem where examples formed by pairs of sequentially close residues are considered. Most of the zinc-binding sites contain at least one of such pairs, which in the following will be named *semi-patterns*. We developed a semi-pattern SVM trained to predict the zinc-binding attitude of a full semi-pattern. A traditional 1D SVM predictor was employed to account for the isolated ligands, and the final prediction for a given residue was computed by a gating network combining the probability of belonging to a zinc-binding semi-pattern and that of being an isolated ligand. In the following we will refer to the learning architecture as *SP-SVM *in order to stress the importance of the semi-pattern prediction as well as the role of the SVM components.

The method was tested on a representative non-redundant set of zinc-binding protein chains in order to assess its generalization power on new chains. Two evaluation procedures were employed, a full leave-one-out procedure on a subset with pairwise HSSP-value up to five, and a k-fold cross validation procedure guaranteeing that no test chain was remotely homologous with respect to any chain in the training set (see details in Results). This second test is a stronger requirement with respect to other common approaches to remove redundancy. A significant improvement over the traditional 1D prediction approach was observed. We additionally used the trained predictor to analyze the entire human proteome and observed a good agreement with previous, manually curated, annotations.

## Results and discussion

### PDB data preparation

A data set of high-quality annotated chains was extracted from the Protein Data Bank (PDB) [23] by selecting all the structures deposited in the PDB at June 2005 and containing at least one zinc ion in the coordinate file. Structures binding zinc spuriously because of experimental settings (e.g. high zinc concentration in the crystallization buffer) were removed. Homologs were removed, by retaining only one representative chain. This procedure resulted in a set of 305 unique chains. Amino acids binding to the zinc ion(s) were detected using a threshold of 3 Å for the distance between the metal and the protein donor atoms. In order to provide negative examples of non metal-binding proteins, an additional set was generated by performing a single run of UniqueProt [18] with zero HSSP-value on PDB entries that are not metalloproteins. We thus obtained a second data set of 2,369 chains. Zinc-binding chains whose structure had been solved in the apo (i.e. without metal) form were removed from the ensemble of non-metalloproteins. We computed multiple alignment profiles for all chains using PSI-Blast [24] on the non-redundant (nr) NCBI protein database. In order to reduce noise in the training data we ignored residues whose profile had a relative weight less than 0.015, indicating that too few sequences had aligned at that position. This also allowed to discard poly-histidine tags which are attached at either the N- or C-terminus of some chains in the PDB, as a result of protein engineering aimed at making protein purification easier.

### Analysis of zinc-binding sites

The choice of predicting zinc-binding sites by modelling semi-patterns was motivated by an extensive analysis of the characteristics of the sites, which we briefly report in this section.

Zinc-binding sites of zinc metalloenzymes are traditionally divided into two main groups [6]: catalytic (if the ions bind a molecule directly involved in a reaction) and structural (stabilizing the folding of the protein but not involved in any reaction). In addition, zinc may influence quaternary structure; we consider these cases as belonging to a third site type (interface), which also lacks a catalytic role. Site types can be heuristically correlated to the number of coordinating residues in the same chain. The distribution of site types obtained in this way is reported in Table 1.

**Table 1:** Distribution of zinc site types

# Coordinating Residues	# sites	# chains									
1 (Zn1)	37	20									
2 (interface – Zn2)	65	53									
3 (catalytic – Zn3)	123	106									
4 (structural – Zn4)	239	175									

Any	464	305									

Site types	{1,2}	{1,3}	{1,4}	{2,3}	{2,4}	{3,4}	{1,2,3}	{1,2,4}	{1,3,4}	{2,3,4}	{1,2,3,4}

# chains	14	9	3	21	4	8	7	1	0	2	0

Top: Distribution of site types (according to the number of coordinating residues in the same chain) in the 305 zinc-chain data set. The second column is the number of sites for each site type; the third column is the number of chains having at least one site of the type specified in the row. Bottom: Number of chains containing multiple site types. The second row gives the number of chains that contain at least one site for each of the types specified in the first row.

**Figure 1:**
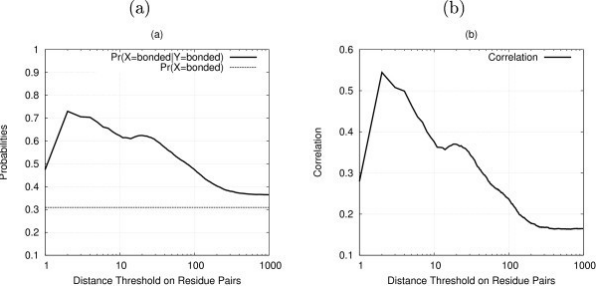
**Correlation between zinc-binding residues**. (a) Probabilities of zinc binding for a given residue: prior and conditioned on the presence of another zinc binding residue within a certain separation, (b) Correlation between the targets of pairs of residues within a given distance.

Table 2 reports the observed binding frequencies grouped by amino acid type and site type. As expected, cysteine, histidine, aspartic acid and glutamic acid are the only residues that bind zinc with a high enough frequency. It is interesting to note that such residues show different binding attitudes with respect to the site type. While cysteines are mainly involved in structural sites and histidines participate to both Zn4 and Zn3 sites with similar frequency, aspartic and glutamic acids are much more common in catalytic sites. The fact that multiple residues coordinate a single zinc ion implies that there is a strong correlation between the bonding state of residues within a given protein chain. Such correlation is often tied to the sequence separation between residues, as many binding sites contain pairs or sequentially close residues. We empirically measured the zinc-bonding state correlation between the residues in our chains. In Figure 1(a) the prior probability of zinc-binding for a residue is compared to the same probability conditioned on the presence of another zinc-binding residue within a certain separation, for different values of the separation threshold. Figure 1(b) reports the correlation coefficient between the bonding state of pairs of residues, again varying the separation threshold between them. Both curves show a very similar behavior, with the highest peak for a distance of less then three residues, and a small one for a distance of around twenty residues. It can be noted that correlation tends to a non zero residual asymptotic value as distance grows, quantifying the contribution due to the fact of belonging to the same chain.

**Table 2:** Amino acid statistics on zinc sites

Site type	Zn4			Zn3			Zn2			Zn1			All
Amino acid	*N*_*a*_	*f*_*a*_	*f*_*s*_	*N*_*a*_	*f*_*a*_	*f*_*s*_	*N*_*a*_	*f*_*a*_	*f*_*s*_	*N*_*a*_	*f*_*a*_	*f*_*s*_	*N*_*a*_

C	663	69.3	91.8	45	12.2	6.2	10	7.7	1.4	4	10.8	0.6	722
H	220	23.0	45.7	194	52.6	40.3	59	45.4	12.3	8	21.6	1.7	481
D	48	5.0	27.6	83	22.5	47.7	30	23.1	17.2	13	35.1	7.5	174
E	18	1.9	17.5	46	12.5	44.7	28	21.5	27.2	11	29.7	10.7	103
N	5	0.5	83.3	0	0.0	0.0	1	0.8	16.7	0	0.0	0.0	6
Q	2	0.2	33.3	1	0.3	16.7	2	1.5	33.3	1	2.7	16.7	6

Total	956	100	-	369	100	-	130	100	-	37	100	-	1492

Statistics over the 305 zinc chains (464 binding sites) divided by amino acid and site type. *N*_*a *_is the amino acid occurrence number in corresponding site type; *f*_*a *_is the observed percentage of each amino acid in a given site type; *f*_*s *_is the observed percentage of each site type for a given amino acid. All is the total number of times a given amino acid binds zinc in general.

### Patterns of binding sites

**Figure 2:**
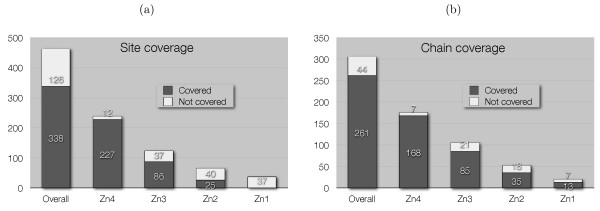
**[CHDE] x(0–7) [CHDE] coverage**. Site (a) and chain (b) coverage for the [CHDE] x(0–7) [CHDE] semi-pattern, both overall and divided by site type.

Metal binding sites can be described by patterns characterized by the type of residues coordinating the same ion and their sequence separation. Table 3 reports the most commonly occurring zinc-binding patterns together with their number of occurrences within our data set. Note that more than one pattern can match at a given site. Many of these sites, especially the structural ones, contain pairs of coordinating residues whose sequence separation is within seven residues. Such pairs are identified by an S (standing for "short") in the Type column of Table 3, as opposed to L (standing for "long") which identifies pairs of residues with a sequence separation of at least eight residues. In the following, a pattern formed by a single pair of nearby coordinating residues is called a *semi-pattern*. Most structural sites consist of two semi-patterns whose distance ranges between 8 and 29 (the SLS Type in Table 3). Catalytic sites typically contain a semi-pattern and a single residue (SL or LS Type). Finally, interface sites are observed as a single semi-pattern in each chain. Figure 2 shows the fraction of sites (a) and zinc chains (b) containing at least once the semi-pattern [CHDE] x(0–7) [CHDE]. These observations suggest a partial solution to the relational auto-correlation problem based on binary classification of semi-patterns to predict binding sites.

**Table 3:** Zinc binding site patterns

Binding Site Patterns	*N*	Type
[CHDE] x(·) [CHDE] x(·) [CHDE] x(·) [CHDE]	232	
[CH] x(·) [CH] x(·) [CH] x(·) [CH]	196	
[CHDE] x(0–7) [CHDE] x(·) [CHDE] x(0–7) [CHDE]	161	
[CHDE] x(0–7) [CHDE] x(> 7) [CHDE] x(0–7) [CHDE]	141	SLS
[CHDE] x(·) [CHDE] x(·) [CHDE]	122	
[C] x(·) [C] x(·) [C] x(·) [C]	85	
[CHDE] x(·) [CHDE]	62	
[CHDE] x(0–7) [CHDE] x(> 7) [CHDE]	55	SL
[CH] x(·) [CH] x(·) [CH]	37	
[CHDE] x(> 7) [CHDE] x(0–7) [CHDE]	24	LS
[CH] x(·) [CH]	21	
[CHDE] x(0–7) [CHDE] x(> 7) [CHDE] x(> 7) [CHDE]	17	SLL
[CHDE] x(> 7) [CHDE] x(0–7) [CHDE] x(0–7) [CHDE]	16	LSS
[DE] x(·) [DE]	15	
[DE] x(·) [DE] x(·) [DE]	10	
[CHDE] x(> 7) [CHDE] x(> 7) [CHDE] x(0–7) [CHDE]	10	LLS
[CHDE] x(0–7) [CHDE] x(0–7) [CHDE] x(> 7) [CHDE]	8	SSL
[DE] x(·) [DE] x(·) [DE] x(·) [DE]	1	

Binding site patterns ordered by number of matches, *N*, in the 464 sites. Note that more than one pattern can match at a given site, thus the total number of matches is greater than the total number of sites. Square brackets denote alternative binding residues, x(·) denotes a sequence of residues of an arbitrary length, x(*n *- *m*) denotes a sequence of between *n *and *m *residues, x(> *n*) denotes a sequence of more than *n *residues. The type column highlights some common binding site patterns: S stands for "short" and refers to x(0–7), L stands for "long" and refers to x(> 7).

### Evaluation of SVM-based predictors

A traditional 1D SVM predictor was compared to the full SP-SVM architecture, in order to assess the significance of the proposed approach. While aspartic and glutamic acids coordinate zinc ions less frequently than cysteines and histidines (see Table 2), they are far more abundant in protein chains. This yielded an extremely unbalanced data set, and forced us to initially focus on cysteine and histidine residues only (we will refer to such predictor as *SP*_*CH*_-*SVM*). Moreover, we labelled a [CH] x(0–7) [CH] semi-pattern as positive if both candidate residues bound a zinc ion, even if they were not actually binding the same ion. Preliminary experiments showed this to be a better choice than considering such a case as a negative example, allowing to recover a few positive examples, especially for semi-pattern matches with longer gaps. Model selection was performed by a stratified 4-fold cross validation procedure on the full data set, aimed at tuning Gaussian kernel width, *C *regularization parameter, window size and parameters of the sigmoids of the gating network. Due to the strong unbalance of the data set, accuracy is not a reliable measure of performance. We used the area under the recall-precision curve (AURPC) for both model selection and final evaluation, as it is especially suitable for extremely unbalanced data sets. We also computed the area under the ROC curve (AUC) to further assess the significance of the results.

Generalization performances of the best models for the local predictor and the gating network were assessed with two different procedures. First, we evaluated generalization over non-homologous chains. We repeatedly run UniqueProt [18] with HSSP-value equal to five starting from the full data set and stopping when then program found only clusters of singletons, thus assuring that no two chains had an HSSP-value greater than the threshold. We then run a full leave-one-out (LOO) procedure on the resulting data set, which consisted of 230 zinc-binding chains and 1,949 negative ones. Second, we evaluated generalization over chains which had no remote homologue in the training set. To this aim, we employed a stratified five fold cross validation (CV) procedure on the full data set. Few (38) non-metalloprotein chains were removed in this procedure as they lacked the information about SCOP [19] classification, which prevented us from assigning them to the correct CV fold. In fact, we distributed protein chains over the CV folds by ensuring that two chains having a zinc-binding domain belonging to the same SCOP [19] superfamily always appeared in the same CV fold, and two free chains (which were employed as negative examples) having a domain in the same SCOP superfamily also appeared in the same CV fold. In this way, we measure generalization across different super-families, a setting in which not even remote homology modelling techniques could be successfully applied for prediction. Note that by *k*-fold cross-validation we mean splitting the data in *k *subsets (commonly called folds) and using one of them in turn for testing. The term "fold" in SCOP has a totally different meaning.

**Figure 3:**
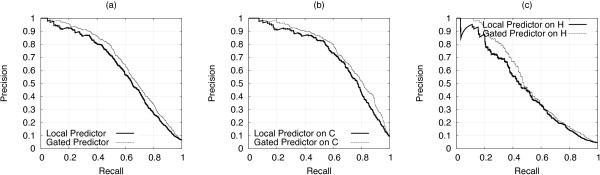
**LOO: local vs gated predictor at a residue level**. LOO procedure: residue level recall-precision curves for the best local CH-SVM and gated SP_*CH*_-SVM predictors, (a) cysteines and histidines together, (b) cysteines only, (c) histidines only.

In the LOO procedure, the local predictor and the gating network obtained an AURPC equal to 0.590 and 0.633 respectively. Figure 3 reports full recall-precision curves, showing that the gating network consistently outperforms the local predictor. While cysteines are far better predicted than histidines, both predictions are improved by the use of the gating network. AUC values were 0.895 ± 0.007 and 0.914 ± 0.006 for local predictor and gating network respectively, where the confidence intervals are the standard error of the Wilcoxon-Mann-Whitney statistic, confirming that the gating network attains a significant improvement over the local predictor.

**Figure 4:**
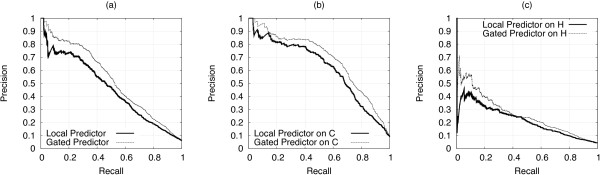
**CV: local vs gated predictor at a residue level**. CV procedure: residue level recall-precision curves for the best local CH-SVM and gated SP_*CH*_-SVM predictors, (a) cysteines and histidines together, (b) cysteines only, (c) histidines only.

The CV procedure gave similar results. The local predictor and the gating network obtained an AURPC equal to 0.428 and 0.500 respectively, and full recall-precision curves are shown in Figure 4. Both methods are able to generalize over non remotely-homologue chains, and the performance of the gating network is still significantly higher than that of the local predictor, as confirmed by AUC values of 0.890 ± 0.006 and 0.867 ± 0.007 respectively.

**Figure 5:**
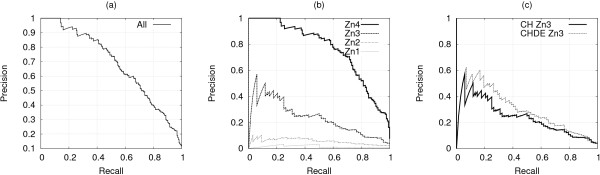
**LOO: SP_***CH***_-SVM predictions at a protein level**. LOO procedure: protein level recall-precision curves for the best SP_*CH*_-SVM. (a) all chains together, (b) chains divided by zinc site type, (c) chains with Zn3 sites, comparison with the best SP_*CHDE*_-SVM.

**Figure 6:**
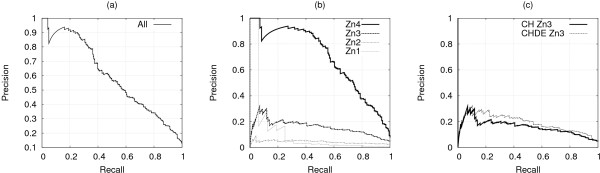
**CV: SP_***CH***_-SVM predictions at a protein level**. CV procedure: protein level recall-precision curves for the best SP_*CH*_-SVM. (a) all chains together, (b) chains divided by zinc site type, (c) chains with Zn3 sites, comparison with the best SP_*CHDE*_-SVM.

Protein-level predictions were computed by requiring that at least three residues within the chain were predicted to bind zinc with a given probability, as computed by the gating network (Eq. (2)). By varying such probability we obtained a recall-precision curve at the chain level. Figures 5(a) and 6(a) report the curves obtained by using the best gated predictor for the LOO and CV procedure respectively, while Figures 5(b) and 6(b) show the results separately for chains containing different binding site types. As expected, Zn4 sites were the easiest to predict, being the ones showing the strongest regularities and most commonly containing the [CH] x(0–7) [CH] semi-pattern.

Finally, we investigated the viability of training a predictor for all the four amino acids involved in zinc binding (it will be referred to as *SP*_*CHDE*_-*SVM*), trying to overcome the disproportion issue. On the rationale that binding residues should be well conserved because of their important functional role, we put a threshold on the residue conservation (Pr(*X*)) in the multiple alignment profile in order to consider it as a candidate target. By requiring that Pr(*D*) + Pr(*E*) ≥ 0.8, we more than halved the unbalance in the data set for the local predictor. At the level of semi-patterns, we realized that such a threshold produced a reasonable unbalance only for gap lengths between one and three, and thus decided to ignore semi-patterns containing aspartic or glutamic acid with gaps of different lengths. While global performances were almost unchanged, aspartic acid and glutamic acid alone obtained a value of the AUC of 0.74 ± 0.03 and 0.70 ± 0.06 respectively in the LOO procedure and 0.73 ± 0.03 and 0.65 ± 0.05 in the CV procedure (with respect to the 0.5 baseline), showing that performances are significantly better than random. However, results on these two residues are still preliminary and further work is required to provide a prediction quality comparable to that obtained for cysteines and histidines. It is interesting to note that at the level of chain classification, the only difference that can be noted by using [CHDE] instead of [CH] is an improvement in the performances for the Zn3 binding sites, as shown in Figures 5(c) and 6(c). This is perhaps not surprising given that half of [DE] residues binding zinc are contained in Zn3 sites, as reported in Table 2. The list of protein chains employed in the two experimental settings, together to the splits of the 5-fold cross validation procedure and the model parameters obtained in the tuning phase, are available in the additional file 1.

### Predictions for the human proteome

A bioinformatic analysis of the content of the human proteome in terms of zinc-binding proteins is already available [9]. In that work, putative zinc-binding proteins were identified based on the occurrence of known (from the PDB) zinc-binding patterns together with some sequence similarity around the pattern, following a previously proposed methodology [10]. These results were integrated by those independently obtained by i) text-mining the available annotations of human genes and ii) using Pfam protein domains described as having zinc-binding properties to scan the proteome. These three search approaches cumulatively allowed identification of zinc-binding proteins in the entire PDB with a precision of 78% and a recall of 89% [9]. This strategy is intrinsically limited in that it can exploit thoroughly existing information but cannot predict new binding sites. Nevertheless, when applied to the human proteome, it identified ab. 3,200 human chains that are potentially zinc binding. Of these, 53% were identified independently by all three approaches, and 76% were identified by at least two methods [9]. These results required a significant degree of manual care (e.g. in the selection of Pfam domains to be searched) and contain a certain degree of subjectivity (e.g. due to the fact that several gene annotations are relatively speculative). The present approach, which is fully automated, has a performance on the PDB only slightly worse than that of the manually curated methodology described in [9], while providing the unique opportunity of predicting unprecedented zinc-binding patterns and thus entirely new classes of zinc-proteins, as discussed in detail below.

To meaningfully compare the presently developed SVM-based approach and the above-described published work, the SP-SVM was used to scan the same human proteome version for putative zinc-binding chains. In the present approach a chain is dubbed as zinc-binding if the predictor assigns a probability of being zinc-binding greater than 0.7 to at least three residues in the chain. By doing so, we switch from per-residue prediction (SP-SVM output) to a per-protein prediction. Indeed, the output most relevant for the biologists is the prediction of zinc-binding capabilities at the entire protein level.

The SP_*CH*_-SVM identified 2,833 putative human zinc-binding chains, which constitute the predicted human zinc-proteome. The results obtained employing the *SP*_*CHDE*_-SVM are very similar to those of the SP_*CH*_-SVM, possibly because the comparatively small number of available examples of sites containing aspartic and glutamic acids as ligands limits the training of the machine.

**Figure 7:**
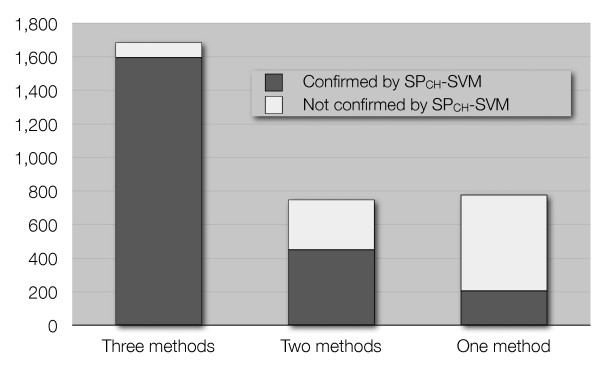
**Predictions on the Human proteome**. Identification of previously detected human zinc-binding chains by the SP_*CH*_-SVM (dark gray: chains retrieved by the predictor, light gray: chains not retrieved by the predictor) as a function of the reliability of the previous results [9].

Comparing the present results with those previously published, we verified that 965 out of 3,207 putative zinc-binding chains were not confirmed by the SP_*CH*_-SVM. However, not all the 3,207 human proteins found in [9] are equally likely to be true zinc-binding chains, and three different qualitative levels of likelihood were identified [9]. Figure 7 shows that the distribution of the chains retrieved by the SP_*CH*_-SVM is in agreement with the results of the previously published work. Only about 350 chains were previously found by two or three independent approaches but have not been retrieved by the SP_*CH*_-SVM. These 350 protein chains mainly comprise (i) chains that have a domain annotated as zinc-binding but lack any metal-binding pattern; (ii) chains that have a pattern composed by only 2 ligands; (iii) chains that contain at least one domain annotated as zinc-binding but not yet structurally characterized (therefore no metal-binding pattern can be associated to these chains). About 100 chains have not been confirmed by the SP_*CH*_-SVM for no obvious reason, corresponding to about 3% of the previously predicted human zinc-proteome.

**Figure 8:**
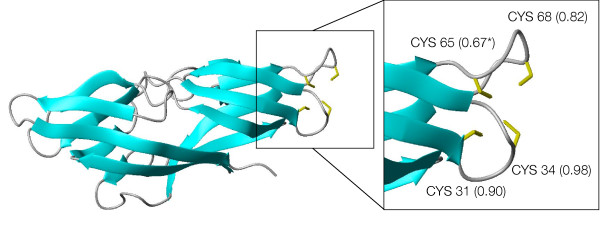
**Structural model of [RefSeq:NP_060357.1]**. Structural model of the hypothetical human protein [RefSeq:NP_060357.1]. The inset contains a close-up of the predicted zinc-binding site, with the side-chains of the putative ligands shown as yellow sticks. Numbers in the inset report the level of confidence estimated by the predictor. The value for Cys65 is slightly below the threshold adopted in this work (0.7).

**Figure 9:**
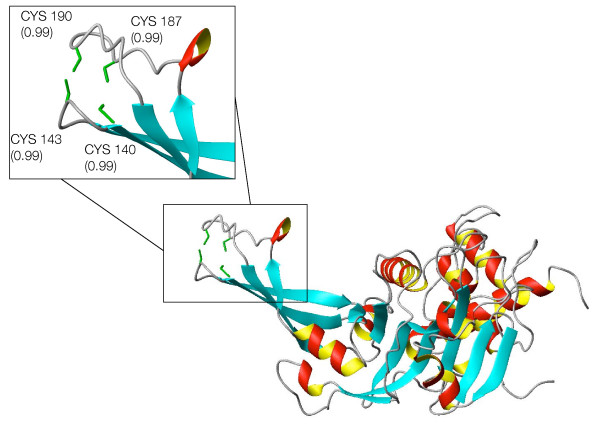
**Structural model of [RefSeq:XP_374396] UCH domain**. Structural model of the UCH domain of the human [RefSeq:XP.374396] protein, a candidate member of the UBP family. The inset contains a close-up of the predicted zinc-binding site, with the side-chains of the putative ligands shown as green sticks. Numbers in the inset report the level of confidence estimated by the predictor.

About 600 proteins not detected in [9] are predicted to be zinc-binding proteins by the SP_*CH*_-SVM. This group comprises some false positives like the tumor necrosis factor receptors or keratin associated proteins which use the predicted zinc-binding cysteines to make disulphide bridges, as well as some possible true positives previously undetected. As an example, a potential zinc-binding site was found in a chain annotated as hypothetical and functionally uncharacterized ([RefSeq:NP_060357.1]). For this chain it was possible to build a 3D-model on the basis of the X-ray structure of the Plasmodium falciparum homologue ([PDB:1ZSO] [20]). The alignment of these two sequences shows that the Plasmodium falciparum protein does not present any potential ligand corresponding to the predicted binding cysteines (CX(2)CX(33)C), and in fact the available structure does not contain any metal. The theoretical structural model of the human protein (Figure 8) shows that these three cysteines are close in space. The metal-binding pattern probably involves another cysteine which was predicted with a probability (0.67) only slightly below the threshold. Homologues of these proteins are only found in eukaryota. The metal-binding pattern is conserved (additional file 2), except in sequences from Alveolata (Plasmodium, Cryptosporidium, Theileria). Another case worth mentioning is that of about 50 human chains annotated as ubiquitin-specific protease and predicted to bind zinc with a CX(2)CX(43–50)CX(2)C pattern. All these proteins are involved in the deubiquitination process and belong to the same family (the UBP family), which contains highly divergent sequences. Their catalytic domain has been structurally characterized and appears similar to an extended right hand, ready to receive the substrate [21]. 3D homology models of these 50 chains confirm their potential capability of binding zinc. As shown in Figure 9 the potential zinc-binding pattern falls at the tip of the fingers of the hand, a region that is directly involved in the interaction with the substrate [21]. The zinc ion may thus stabilize the structure in this peripheral part of the chain, indirectly contributing to the interaction with ubiquitin. A structure of bovine UBP41 released after preparation of this work confirmed the prediction of above zinc-binding site ([PDB:2HD5] [22]). The zinc-binding site is next to the region of interaction of this enzyme with ubiquitin.

Finally, it must be noted that in some cases the SVMs do not predict all the ligands in the structure with a high probability but can predict only a part of the pattern or include erroneous residues in the pattern. An explicative example is the binding-site prediction for the ADAM-TS family. This family, which has not yet been structurally characterized, comprises Zn-dependent endopeptidases using the HX(3)HX(5)H motif to bind the catalytic zinc ion. For all these chains the SVMs predicted the first two histidines as ligands with a high probability (more than 0.7) while the third histidine is often predicted with very low values (average value = 0.32). Chain-level comparisons between SP_*CH*_-SVM and results in [9] are available in the additional file 3.

## Conclusion

In the present work we have described a novel approach based on SVMs to the prediction of zinc-binding capabilities at the level of an entire proteome. The method has been trained using the structures available in the PDB where zinc was bound in a physiologically relevant manner. This should maximize, but cannot guarantee, that the properties predicted are relevant also *in vivo *and not just *in vitro*. However, due to the complexity of the processes controlling the insertion of metal cofactors in proteins and, in particular, due to the fact that they are under kinetic rather than thermodynamic control, it is not possible to exclude that a protein predicted here to be zinc-binding will *in vivo *bind other metal ions (e.g. iron, copper). With all these caveats in mind, the present approach constitutes a highly automated tool for the identification of metalloproteins, which provides results of comparable quality with respect to highly manually refined predictions. In addition, it permits the identification of unprecedented metal sites, providing important hints for the work of experimentalists. The performance of the proposed method was evaluated on strong non-redundancy tests showing a significant improvement due to correlation modelling. The present SVMs exploit well the occurrence in metal-binding sites of cysteine and histidine residues, while there is room for improving the performance with respect to sites containing aspartic and glutamic acid residues.

## Methods

### Prediction using SVM

Many applications of machine learning to 1D prediction tasks use a simple vector representation obtained by forming a window of flanking residues centered around the site of interest. Evolutionary information is incorporated in this representation by computing multiple alignment profiles [25]. In this approach, each example is represented as a vector of size *d *= (2*k *+ 1)*p*, where *k *is the size of the window and *p *the size of the position specific descriptor. In this paper we developed a learning architecture which expands such representation in order to address the relational auto-correlation problem described in the previous paragraph. A *local predictor *based on SVM [26-28] uses the standard window representation for classifying the zinc-binding state of individual residues. Multiple alignment profiles are enriched by two indicators of profile quality, namely the entropy and the relative weight of gapless real matches to pseudocounts. An additional flag is included to mark positions ranging out of the sequence limits, resulting in an all-zero profile. We thus obtain a position specific descriptor of size *p *= 23. The correlation between nearby residues is modeled by an SVM *semi-pattern predictor*, trained to predict the bonding state of pairs of residues close in sequence. A candidate semi-pattern is a pair of residues separated by a gap of *δ *residues, with *δ *ranging from zero to seven. The task is to predict whether the semi-pattern is part of a zinc-binding site. Each example is represented by a window of local descriptors (based on multiple alignment profiles) centered around the semi-pattern, including the gap between the candidate residues. An ad-hoc *semi-pattern kernel *(*K*_*sp*_) measuring the similarity between two semi-patterns was developed in the following way: given two vectors *x *and *z*, of size *d*_*x *_and *d*_*z*_, representing semi-patterns with gap length *δ*_*x *_and *δ*_*z *_respectively,

Ksp(x,z)=〈x1w,z1w〉+〈xdx−wdx,ydz−wdz〉+ Kgap(xw+1δxp+w,zw+1δzp+w)     (1)
 MathType@MTEF@5@5@+=feaafiart1ev1aaatCvAUfKttLearuWrP9MDH5MBPbIqV92AaeXatLxBI9gBaebbnrfifHhDYfgasaacH8akY=wiFfYdH8Gipec8Eeeu0xXdbba9frFj0=OqFfea0dXdd9vqai=hGuQ8kuc9pgc9s8qqaq=dirpe0xb9q8qiLsFr0=vr0=vr0dc8meaabaqaciaacaGaaeqabaqabeGadaaakeaafaqadeGabaaabaGaem4saS0aaSbaaSqaaiabdohaZjabdchaWbqabaGccqGGOaakcqWG4baEcqGGSaalcqWG6bGEcqGGPaqkcqGH9aqpdaaadeqaaiabdIha4naaDaaaleaacqaIXaqmaeaacqWG3bWDaaGccqGGSaalcqWG6bGEdaqhaaWcbaGaeGymaedabaGaem4DaChaaaGccaGLPmIaayPkJaGaey4kaSYaaaWabeaacqWG4baEdaqhaaWcbaGaemizaq2aaSbaaWqaaiabdIha4bqabaWccqGHsislcqWG3bWDaeaacqWGKbazdaWgaaadbaGaemiEaGhabeaaaaGccqGGSaalcqWG5bqEdaqhaaWcbaGaemizaq2aaSbaaWqaaiabdQha6bqabaWccqGHsislcqWG3bWDaeaacqWGKbazdaWgaaadbaGaemOEaOhabeaaaaaakiaawMYicaGLQmcaaeaacqGHRaWkcqqGGaaicqWGlbWsdaWgaaWcbaGaem4zaCMaemyyaeMaemiCaahabeaakmaabmaabaGaemiEaG3aa0baaSqaaiabdEha3jabgUcaRiabigdaXaqaaGGaciab=r7aKnaaBaaameaacqWG4baEaeqaaSGaemiCaaNaey4kaSIaem4DaChaaOGaeiilaWIaemOEaO3aa0baaSqaaiabdEha3jabgUcaRiabigdaXaqaaiab=r7aKnaaBaaameaacqWG6bGEaeqaaSGaemiCaaNaey4kaSIaem4DaChaaaGccaGLOaGaayzkaaaaaiaaxMaacaWLjaWaaeWaaeaacqaIXaqmaiaawIcacaGLPaaaaaa@7F6B@

where vij
 MathType@MTEF@5@5@+=feaafiart1ev1aaatCvAUfKttLearuWrP9MDH5MBPbIqV92AaeXatLxBI9gBaebbnrfifHhDYfgasaacH8akY=wiFfYdH8Gipec8Eeeu0xXdbba9frFj0=OqFfea0dXdd9vqai=hGuQ8kuc9pgc9s8qqaq=dirpe0xb9q8qiLsFr0=vr0=vr0dc8meaabaqaciaacaGaaeqabaqabeGadaaakeaacqWG2bGDdaqhaaWcbaGaemyAaKgabaGaemOAaOgaaaaa@3106@ is the sub-vector of *v *that extends from *i *to *j*, and *w *= (*k *+ 1)*p*. The first two contributions compute the dot products between the left and right windows around the semi-patterns, included the two candidate residues, whose sizes do not vary regardless of the gap lengths. *K*_*gap *_is the kernel between the gaps separating the candidate residues, and is computed as:

Kgap(u,v)={Kμgap(u,v)+〈u,v〉if|u|=|v|Kμgap(u,v)otherwise
 MathType@MTEF@5@5@+=feaafiart1ev1aaatCvAUfKttLearuWrP9MDH5MBPbIqV92AaeXatLxBI9gBaebbnrfifHhDYfgasaacH8akY=wiFfYdH8Gipec8Eeeu0xXdbba9frFj0=OqFfea0dXdd9vqai=hGuQ8kuc9pgc9s8qqaq=dirpe0xb9q8qiLsFr0=vr0=vr0dc8meaabaqaciaacaGaaeqabaqabeGadaaakeaacqWGlbWsdaWgaaWcbaGaem4zaCMaemyyaeMaemiCaahabeaakmaabmaabaGaemyDauNaeiilaWIaemODayhacaGLOaGaayzkaaGaeyypa0ZaaiqabeaafaqaaeGacaaabaGaem4saS0aaSbaaSqaaGGaciab=X7aTjabdEgaNjabdggaHjabdchaWbqabaGcdaqadaqaaiabdwha1jabcYcaSiabdAha2bGaayjkaiaawMcaaiabgUcaRmaaamqabaGaemyDauNaeiilaWIaemODayhacaGLPmIaayPkJaaabaGaeeyAaKMaeeOzay2aaqWaaeaacqWG1bqDaiaawEa7caGLiWoacqGH9aqpdaabdaqaaiabdAha2bGaay5bSlaawIa7aaqaaiabdUealnaaBaaaleaacqWF8oqBcqWGNbWzcqWGHbqycqWGWbaCaeqaaOWaaeWaaeaacqWG1bqDcqGGSaalcqWG2bGDaiaawIcacaGLPaaaaeaacqqGVbWBcqqG0baDcqqGObaAcqqGLbqzcqqGYbGCcqqG3bWDcqqGPbqAcqqGZbWCcqqGLbqzaaaacaGL7baaaaa@7229@

with

Kμgap(u,v)=〈p|u|∑i=1|u|/pu(i−1)p+1ip,p|v|∑i=1|v|/pv(i−1)p+1ip〉
 MathType@MTEF@5@5@+=feaafiart1ev1aaatCvAUfKttLearuWrP9MDH5MBPbIqV92AaeXatLxBI9gBaebbnrfifHhDYfgasaacH8akY=wiFfYdH8Gipec8Eeeu0xXdbba9frFj0=OqFfea0dXdd9vqai=hGuQ8kuc9pgc9s8qqaq=dirpe0xb9q8qiLsFr0=vr0=vr0dc8meaabaqaciaacaGaaeqabaqabeGadaaakeaacqWGlbWsdaWgaaWcbaacciGae8hVd0Maem4zaCMaemyyaeMaemiCaahabeaakmaabmaabaGaemyDauNaeiilaWIaemODayhacaGLOaGaayzkaaGaeyypa0ZaaaWabeaadaWcaaqaaiabdchaWbqaamaaemaabaGaemyDauhacaGLhWUaayjcSdaaamaaqahabaGaemyDau3aa0baaSqaamaabmaabaGaemyAaKMaeyOeI0IaeGymaedacaGLOaGaayzkaaGaemiCaaNaey4kaSIaeGymaedabaGaemyAaKMaemiCaahaaaqaaiabdMgaPjabg2da9iabigdaXaqaamaaemaabaGaemyDauhacaGLhWUaayjcSdGaei4la8IaemiCaahaniabggHiLdGccqGGSaaldaWcaaqaaiabdchaWbqaamaaemaabaGaemODayhacaGLhWUaayjcSdaaamaaqahabaGaemODay3aa0baaSqaamaabmaabaGaemyAaKMaeyOeI0IaeGymaedacaGLOaGaayzkaaGaemiCaaNaey4kaSIaeGymaedabaGaemyAaKMaemiCaahaaaqaaiabdMgaPjabg2da9iabigdaXaqaamaaemaabaGaemODayhacaGLhWUaayjcSdGaei4la8IaemiCaahaniabggHiLdaakiaawMYicaGLQmcaaaa@7A9F@

*K*_*μgap *_computes the dot product between the average position specific descriptors within each gap, and if the two gaps have same length, the full dot product between the descriptors in the gaps is added.

We employ a Gaussian kernel on top of both the linear kernel of the local predictor and the semi-pattern kernel (Eq. (1)). To get a better performance, we combine the single output from the local predictor on a given residue and the (possibly empty) set of outputs from the semi-pattern based predictor by a *gating network*. In order to combine two predictors, it is preferable to convert their SVM functional margins into conditional probabilities using the sigmoid function approach suggested in Platt [29]:

*P*(*Y *= 1|*x*) = 1/(1 + exp (-*Af*(*x*)-*B*)) where *f*(*x*) is the SVM output for example *x *and sigmoid slope (*A*) and offset (*B*) are parameters to be learned from data. The probability *P*(*Y*_*b *_= 1|*x*) that a single residue binds zinc can now be computed by the following gating network:

*P*(*Y*_*b *_= 1|*x*) = *P*(*Y*_*s *_= 1|*x*) + (1 - *P*(*Y*_*s *_= 1|*x*))*P*(Y_*l *_= 1|*x*)     (2)

where *P*(*Y*_*l *_= 1|*x*) is the probability of zinc binding from the local predictor, while *P*(*Y*_*s *_= 1|*x*) is the probability of *x *being involved in a positive semi-pattern, approximated as the maximum between the probabilities for each semi-pattern *x *is actually involved in.

### Validation through homology modelling

We attempted to model the 3D structure of all the human chains retrieved by the present SP_*CH*_-SVM but not reported in the literature or previously predicted to be zinc-binding. Appropriate templates were looked for in the PDB, by searching for proteins of known structure having a sequence identity greater than 30% to the target. Structural models were built using the program Modeller-6v2 [30]. The input alignment for Modeller was obtained with ClustalW [31].

## Availability and requirements

**Project Name**: Zinc Finder

**Project home page**: 

**Operating system(s)**: Platform independent

**Programming language**: c++

**Other requirements**: c++ compiler

**License**: GNU GPL

**Any restrictions to use by non-academics**: none

## Authors' contributions

AP developed the semi-pattern kernel and the full architecture and run all final experiments. CA provided insights into the characteristics of zinc-binding sites which inspired the method and conducted the detailed analysis of predictions on the Human proteome. SM performed the analysis of zinc binding sites and residues correlation and run extensive preliminary experiments. AR an PF wrote background and conclusions and provided insights and suggestions both in the development of the predictor and in the analysis and validation procedures.

## Supplementary Material

Additional File 1 **SVM evaluation data**. List of protein chains employed in the experiments, splits of the 5-fold cross validation procedure, model parameters obtained in the tuning phase. Click here for file

Additional File 2 **[RefSeq:NP_060357.1] alignment**. Metal binding pattern alignment for [RefSeq:NP_060357.1]. Click here for file

Additional File 3 **SP_***CH***_-SVM vs previous methods**. Comparisons at a chain level between predictions of the SP_*CH*_-SVM and results published in Andreini et al., J. Proteome Research, 5(1):196–201, 2006. Click here for file
